# Primary hepatic angiosarcoma: Treatment options for a rare tumor

**DOI:** 10.18632/oncoscience.602

**Published:** 2024-05-20

**Authors:** Gregory L. Guzik, Ankit Mangla

**Affiliations:** ^1^Department of Internal Medicine, University Hospitals Cleveland Medical Center, Cleveland, OH 44106, USA; ^2^Department of Hematology and Oncology, University Hospitals Seidman Cancer Center, Cleveland, OH 44106, USA; ^3^Department of Hematology and Oncology, Case Western Reserve University School of Medicine, Cleveland, OH 44106, USA; ^4^Case Comprehensive Cancer Center, Cleveland, OH 44106, USA

**Keywords:** angiosarcoma, liver angiosarcoma, immunotherapy

Angiosarcoma is a mesenchymal tumor that arises from the endothelium of blood or lymphatic vessels. Primary hepatic angiosarcoma (PHA) is the most common mesenchymal tumor of the liver, yet very rare. In our analysis of the National Cancer Database (2004–2014), the incidence of PHA was only 0.29% compared to hepatocellular carcinoma (HCC), which is the most common epithelial tumor of the liver [1]. A high index of suspicion is needed to diagnose PHA. The clinical presentation of PHA is very similar to that of hepatocellular carcinoma (HCC). In a patient with liver cirrhosis presenting with a liver mass, typical imaging findings are usually sufficient to diagnose HCC [2]. Where a biopsy is seldom performed to establish the diagnosis of HCC, the diagnosis of PHA is exclusively based on pathologic confirmation. The prognosis of patients with PHA is much worse compared to those with HCC (1.9 versus 10.3 months, adjusted hazard ratio (aHR) 2.41, 95% Confidence Interval (CI): 2.1–2.77, *p* < 0.0001). Hence, diagnosing PHA at the outset is critical to determine prognosis and direct the correct treatment.

PHA often presents as a multicentric disease. However, in patients with singular lesions or oligometastatic disease, surgical resection should be pursued. In patients with localized extremity or retroperitoneal soft-tissue sarcoma (STS), surgical resection is the primary treatment of choice. Surgical resection for localized visceral sarcomas resulting in a microscopic negative margin (R0 resection) is associated with the best survival outcome. Although only 14.6% of patients in the NCDB database received surgical resection, the overall survival (OS) of these patients was significantly better than those who did not receive surgery (7.7 vs. 1.8 months, aHR-0.23, 95% CI: 0.15–0.37, *P* < 0.0001) [1]. Cytotoxic chemotherapy is the most commonly used systemic treatment for patients with multifocal or metastatic PHA. In patients with angiosarcoma, taxane-based regimens have response rates similar to those of anthracycline-based regimens. The commonly used regimen is weekly paclitaxel at 80 mg/m2 given on days 1, 8, and 15 of a 28-day cycle [3]. The overall response rate (ORR) at 2, 4, and 6 months was ~20%, and the progression-free survival (PFS) rate at six months was 24%. The ORR from taxanes is comparable to that of doxorubicin-based regimens. The most commonly used anthracycline-based regimen involves the concomitant use of doxorubicin (at 75 mg/m2) and ifosfamide (at 6–9 gm/m2 divided over 3 to 5 days) [4]. The dose of ifosfamide differs according to institutional preferences. In patients with PHA, recipients of cytotoxic chemotherapy have a 56% reduction in risk of death compared to those who do not receive chemotherapy (aHR-0.44, 95% CI: 0.32–0.60, *p* < 0.0001).

Checkpoint inhibitors have also been effective in patients with angiosarcoma, particularly cutaneous angiosarcoma of the head and neck. It is likely because of these patients’ high ultraviolet (UV) signature, which is associated with a higher tumor mutation burden (TMB). A sub-study of the DART (Dual Anti-CTLA-4 and Anti-PD-1 Blockade in Rare Tumors) trial (S1609) reported the outcomes of 16 patients (nine with primary cutaneous and seven with non-cutaneous angiosarcoma) [5]. Overall response rate was 25% (4/16 patients), with three responses recorded for those with cutaneous angiosarcoma. One out of seven patients with non-cutaneous angiosarcoma achieved a confirmed response [5]. The Alliance A091902 trial is a triple-arm study, where patients with paclitaxel-refractory angiosarcoma received nivolumab (480 mg IV Q4 weeks) with cabozantinib (40 mg PO daily) [6]. Of the 23 patients reported at the ASCO 2022 (American Society of Clinical Oncology) meeting, the response was evaluable in 22 patients (13 patients with cutaneous and nine with non-cutaneous angiosarcoma). The ORR was 59% (13 out of 22 patients), and the ORR for non-cutaneous angiosarcoma was 67% (6 out of 9 patients). The synergism between anti-PD1 monotherapy and a vascular endothelial growth factor inhibitor resulted in a higher response rate for non-cutaneous angiosarcoma, which has never been demonstrated in previous studies with checkpoint inhibitors. Hence, this combination holds the best promise in a taxane-refractory setting. The other leg of the trial, randomizing treatment-naïve patients with angiosarcoma between paclitaxel versus nivolumab and paclitaxel, is under evaluation. This leg of the trial will inform the utility of the combination of chemotherapy-immunotherapy approach in angiosarcoma. The results are expected to be presented at ASCO 2024. Neoadjuvant chemotherapy has never shown a survival benefit in patients with STS [7]. However, the promise of anti-PD1 and VEGF-I synergism has reignited the debate of bringing this combination into the neoadjuvant setting for resectable sarcomas, especially angiosarcomas. The premise of neoadjuvant therapy with checkpoint inhibitors lies in the fact that a higher tumor burden helps expand the appropriate T-cell clonal population, which in turn may lead to a durable response. The neoadjuvant approach has proved beneficial in melanoma [8]. The 2-year relapse-free survival (RFS) was 97% in those achieving pathologic complete response (pCR) [9]. In addition, the durability of response obtained from checkpoint inhibitors has been demonstrated in several tumor types, including STS. Hence, it makes sense that a rare yet aggressive tumor-like PHA may benefit from neoadjuvant therapy involving the combination of a checkpoint inhibitor with a VEGF-I followed by surgical resection.

Lastly, in our analysis, we also found that patients treated at an academic center had numerically better survival compared to those treated at non-academic centers (2.9 months vs. 1.9 months, aHR-0.99, 95% CI: 0.74–1.34), *p* = 0.97) [1]. It is a well-established fact that the management of patients with any STS is best determined in a multi-disciplinary setting. Academic centers have access to experts in various disciplines, the ability to perform advanced testing (genomics looking for fusions, mutations, and targets), and the availability of clinical trials, which can lead to better outcomes in patients diagnosed with rare cancers like PHA. To conclude, PHA is a rare yet aggressive mesenchymal tumor of the liver, which requires a multi-disciplinary approach to achieve the best patient outcomes.
